# Mental Health Monitoring for Young People Through Mood Apps: Protocol for a Scoping Review and Systematic Search in App Stores

**DOI:** 10.2196/56400

**Published:** 2024-11-19

**Authors:** Selina Boege, Madison Milne-Ives, Ananya Ananthakrishnan, Cen Cong, Aditya Sharma, David Anderson, Edward Meinert

**Affiliations:** 1 Translational and Clinical Research Institute Newcastle University Newcastle upon Tyne United Kingdom; 2 Centre for Health Technology School of Nursing and Midwifery University of Plymouth Plymouth United Kingdom; 3 Academic Psychiatry Translational and Clinical Research Institute Newcastle University Newcastle upon Tyne United Kingdom; 4 Specialist Adolescent Mood Disorders Service (SAMS) Cumbria, Northumberland, Tyne and Wear NHS Foundation Trust Newcastle upon Tyne United Kingdom; 5 Department of Primary Care and Public Health Imperial College London London United Kingdom; 6 Faculty of Life Sciences and Medicine King's College London London United Kingdom

**Keywords:** digital health, mental health, mood apps, mobile apps, mobile phone

## Abstract

**Background:**

The researchers have used mobile phones to assist in monitoring, analyzing, and managing moods to acquire insight into mood patterns. There is a lack of evidence in their use as clinical tools and interventions, which necessitates a comprehensive review and quality assessment to understand barriers and facilitators for app implementation as an impactful clinical intervention.

**Objective:**

This review aims to (1) provide an overview of the recent evidence on mobile mood-monitoring apps that are intended for facilitating self-management and support of mental health in children, adolescents, and young people; and (2) investigate the quality of publicly available apps.

**Methods:**

The study will first involve a scoping review of the literature on mood-monitoring apps for children, adolescents, and young people followed by an evaluation of features of the apps available in the marketplace. The scoping review will follow the PRISMA-ScR (Preferred Reporting Items for Systematic Reviews and Meta-Analyses extension for Scoping Reviews) guidelines and search 6 databases— Embase, CINAHL, PubMed, ACM Digital Library, Scopus, and Springer LNCS—for relevant studies and reviews published in the last 3 years. The author will then screen the references, extract data from the included studies, and analyze them to synthesize the evidence on mood apps. Next, the Apple App Store and Google Play Store will be searched for mood apps. A total of 2 independent reviewers will screen the apps based on eligibility criteria, and disagreements will be resolved through consensus. The features of the selected apps will then be evaluated using the Mobile Health Index and Navigation framework, and descriptive analysis will be used to synthesize the findings.

**Results:**

Literature search and screening began soon after submission of the protocol and is expected to be completed by September 2024. The app evaluation will be completed by October 2024.

**Conclusions:**

Combined, the scoping literature review and app evaluation will provide an in-depth overview of the most recent scientific evidence related to mood apps and the quality of apps actually available for use.

**International Registered Report Identifier (IRRID):**

PRR1-10.2196/56400

## Introduction

Globally, there has been a surge in mental health concerns over the past decade. In 2019, there were over 970 million reported cases of depression and anxiety disorders worldwide [1]. This is particularly the case for children, adolescents, and young people, who are vulnerable to mental health difficulties as they grow [2]. Simultaneously, the use of smartphones, especially among children, adolescents, and young people, has become ubiquitous, where popularity and ownership have exponentially increased and their potential for health promotion has heralded a new era of digital health products [3,4]. There has been a movement toward technology that embraces the support for mental health through digital solutions [5]. Such digital health apps hold the potential to overcome barriers faced by individuals seeking mental health support. This approach brings health support within reach of diverse users, including those residing in geographically remote or underserved regions and individuals with limited financial resources [6,7]. Interest in these tools by children, adolescents, and young people, who seek assistance through mood-monitoring apps for mental health support, has significantly grown [7]. As children, adolescents, and young people typically exhibit a reluctance to seek professional help and often contend with the high prevalence of mental health issues [8], digital health apps can ease concerns related to the stigma associated with mental health challenges [9].

Mental health is a large area of product development within digital health [10]. Many mental health apps have entered the market with the aim of providing methods for addressing symptoms of mental health conditions, such as depression or anxiety and improving overall quality of life and well-being [11]. A subset of these apps includes mood-monitoring features, often in addition to self-management interventions, such as cognitive behavioral therapy or educational content. Some apps have also been developed with the sole purpose of monitoring mood through tools such as daily mood ratings and free-text journaling. This feature is becoming increasingly popular as it has the potential to help increase self-awareness, improve engagement, and facilitate better diagnosis of mood disorders [12-14].

The landscape of currently available apps is diverse, with an estimate of over 20,000 mental health apps available in app stores [15,16]. Only a small fraction of these apps have undergone empirical or clinical scrutiny, suggesting limited evidence of their effectiveness, with less than 5% of all mental health apps confirmed to have clinical evidence [17]. While published evidence is not required for medical device registration, it plays a significant role in the regulatory approval process and the adoption of medical devices in clinical practice [18]. The lack of a comprehensive understanding of validated mood apps may confuse both users and clinicians in selecting apps to meet their personal and professional needs [19].

Previous research has explored the perspectives of people using mood-tracking apps [13,14]. One study found an overall preference for features of personalization and visualization of previously tracked moods among users, while the lack of app-facilitated recommendations for how to interpret user data to improve mood was seen as a missing element [14]. An and Lee [20] used the Mobile App Rating Scale (MARS) to determine which features should be modified to improve the quality of apps [20]. The review demonstrated that most mood management apps received high scores for functionality and aesthetics, but low scores for subjective quality and specific app characteristics, underlining the need for additional refining of evidence-based principles. Rickard et al [3] used the American Psychiatric Association’s 5-step criteria assessment tool to assess the quality and integrity of the most visible 100 apps for “depression,” “anxiety,” and “mood” on the Google Play Store and Apple App Store with a wider scope of general mental health apps and their function types, for example, mental health promotion, prevention, and intervention. The study reveals that out of 10 included and analyzed apps, only 3 met the criteria for clinical base and ease of use. App quality did not significantly vary across function types, with no correlation between app quality and app visibility. The results highlight the risks of insufficient synopsis of “high quality” mood apps for vulnerable users.

To mitigate risks of misinformed decision-making, missed self-help, or the risk of worsening the user’s emotional well-being, various app assessment hubs like the Mobile Health Index and Navigation Database (MIND), Open mHealth, Beacon, or Mindtools.io were established [21,22]. MIND is a publicly available and cost-free repository comprising more than 650 mental health apps that have been assessed using 105 different criteria, with particular emphasis on accessibility, privacy, evidence-based efficacy, user engagement, and therapeutic objectives. Users have the capability to apply various filters to find apps that match their specific requirements and can access detailed information on apps across 9 distinct categories. The apps listed on MIND undergo regular updates, ensuring that the information provided remains current and offers insights into how these apps evolve over time, typically within a span of 100 to 180 days [23].

While there is previous research on the evaluation of quality features of general mental health apps drawn through different measures, common findings indicate that apps offering engaging designs and user-friendly aesthetics are most likely to attract users and improve satisfaction [18]. There is an overall gap in standardized app quality and effectiveness measures on mood apps specifically, app guidance for clinicians and consumers to interpret data, and the improvement of evidence-based mood apps that incorporate consumer preferences [24]. This review aims to bridge this gap by offering an overview of currently available mood-tracking apps on the Google Play Store and Apple App Store as well as the available evidence on mood apps, identifying the strengths and limitations of the apps through a quality evaluation and the gaps in the research through a scoping literature review. The review will focus on mood apps designed for children, adolescents, and young people.

## Methods

### Design

We will use the PRISMA-ScR (Preferred Reporting Items for Systematic Reviews and Meta-Analyses extension for Scoping Reviews) [25] guidelines to guide the search and selection of studies and apps for evaluation (Multimedia Appendix 1). This evaluation will be composed of an app and literature search, screening and app selection, data extraction, data analysis, and data synthesis. The study will begin with a literature review, the results of which will be used to inform a review of features of mood-monitoring apps for young people. The Population, Intervention, Comparator, Outcome, Study (PICOS) framework will be used to develop the search strategy for both the literature review and app search (Table 1) [26,27].

**Table 1:** The Population, Intervention, Comparator, Outcome, Study (PICOS) framework.

Category	Description
Population	Children, adolescents, and young people
Intervention	Mood-monitoring apps (mobile smartphone apps that allow daily mood log and monitoring)
Comparator	—^a^
Outcomes	The primary outcome of the literature review will be an overview of the recent literature on mood-monitoring apps for children, adolescents, and young people. The app search will be an overview of the features and content of mood apps available in the marketplace, updated in the last 3 years (refer to the *Exclusion Criteria* section of phase 2). The secondary outcomes will include a classification of mood apps that test for clinical outcomes and their used measures for the app evaluation and clinical scales used in evaluations for the literature review, as well as a quality evaluation of the 50 most downloaded mood apps.
Study types (only literature review)	Qualitative, quantitative, and mixed methods studies in English that presents studies on mood apps, their review of clinical outcomes, applied measuring methods, and quality assessment of features are eligible for inclusion. Preprints, protocols, abstracts, and meta-analyses with no full-text access and those older than 3 years will be excluded.

^a^Not applicable.

### Scoping Literature Review

#### Search Strategy

The first phase will be a scoping review of the literature on mood-monitoring apps targeted at children, adolescents, and young people. We will search 6 databases—Embase, CINAHL, PubMed, ACM Digital Library, Scopus, and Springer LNCS—using the search structure that includes mental health or mood (Medical Subject Headings OR Keywords) AND mobile apps (Medical Subject Headings OR Keywords) AND children, adolescents, and young people (Medical Subject Headings OR Keywords); Multimedia Appendix 2 provides the full search strings.

#### Inclusion Criteria

The review will include studies evaluating mood-monitoring apps targeted at children, adolescents, and young people. To avoid excluding relevant literature, any app that had mood monitoring as a feature will be included in the review. All types of study designs will be included as long as the study evaluates at least 1 mood-monitoring app. As the scope of the review covers apps targeting children, adolescents, and young people, our search terms will focus on this population; however, to ensure the inclusion of all relevant papers and due to a variety of definitions of “young people” in the literature, we did not specify an age limit.

#### Exclusion criteria

Any study that does not evaluate mood-monitoring apps, including reviews, conference proceedings, reports, posters, and protocols will be excluded. Literature older than 3 years, those in languages other than English, and papers for which the full texts are unavailable will also be excluded from this review. Studies describing a mood-monitoring app without evaluating it will be excluded. Finally, studies of apps that focus on mental health in general without reference to mood monitoring, and those designed for use by health care professionals, will also be excluded.

#### Screening and Paper Selection

References will be exported and stored in the citation management software, EndNote 21 (Clarivate). EndNote will then be used to remove duplicates and conduct automatic screening based on keywords from the search strategy. The titles and abstracts and then the full texts of the remaining papers will be screened by the author (SB) to determine final eligibility. To ensure transparency, the details of this process will be charted in a PRISMA (Preferred Reporting Items for Systematic Reviews and Meta-Analyses) flow diagram.

#### Data Extraction

SB, AS, and CC will read the full text of all included papers and extract data into a predetermined form (Textbox 1). Specific attention will be given to clinical rating scales used to evaluate the apps. Chosen based on clinical consensus, Textbox 2 lists the categories of the rating scales (refer to Multimedia Appendix 3 for a detailed list of typically used validated scales for each category).

**Data extraction items for literature review.**

**Literature**

- Author
- Title
- Year of publication

**App**

- Name of app
- Platform (iOS or Android)
- Allows mood monitoring? (yes or no)
- Targeted age group
- App category
- Focus of app
- Clinical outcomes
- Evaluation measures

**Extraction items of clinical rating scales.**

- Types of clinical rating scales
- Bipolar disorder
- Mania or hypomania
- Depression
- Measures of functioning and quality of life

#### Data Analysis and Synthesis

A descriptive analysis will be used to provide an overview of the research on mood-monitoring apps for children, adolescents, and young people. If applicable, a thematic analysis will be conducted on available qualitative data to better understand user perspectives about the apps.

### App Search and Evaluation

#### Search Strategy

The selection of mood apps will be conducted by searching 2 app marketplaces (Apple App Store and Google Play). A systematic search will be conducted using the keywords “mental health,” “mood,” “depression,” “bipolar disorder,” “mania,” “hypomania,” “mental coach,” and “mood journal”. The executed search through search terms will automatically be displayed by popularity (given the app store’s traffic algorithm) and limited to apps with at least 100,000 downloads. This will ensure that the apps being evaluated are the ones that are most used.

#### Inclusion Criteria

Apps will be eligible if they meet all of the criteria, such as (1) the app’s primary focus is mood tracking, (2) the app operates as self-reporting (no passive sensing), (3) the app has been updated in the last 3 years (due to the disruption of digital mental health support during and after the pandemic), (4) the app is in English, and (5) the app has at least 50 total ratings and reviews on the Apple iOS and Google Play Store. We will include apps regardless of whether they are paid, contain in-app purchases, or are free, and the apps must be designed as a standalone service, without the need for human support (eg, telehealth counselors or web-based service) or additional devices (eg, wearable heart rate monitor).

#### Exclusion Criteria

We will exclude duplicates of apps (if an app is available for both iOS and Android operating systems, we will include the iOS version only) and apps that are not in the English language. Considering the significant change in health app usage during and after the pandemic, we will consider apps that take the disruptive mental health consequences of the COVID-19 pandemic into account and, therefore, exclude apps that have not been updated in the past 3 years [24]. As we focus on mood-tracking apps that focus on the improvement of the user’s well-being, we will exclude any apps that do not provide the mood-tracking feature. To achieve the research objective of offering a holistic overview of existing mood-tracking apps specifically for children, adolescents, and young people, mood apps that are specifically designed for elderly people will also be excluded as they do not fit the population type.

#### Screening and App Selection

All mood apps found through the app marketplaces will be recorded in a Microsoft Excel document and duplicates will be removed, including Android apps that also have an iOS version. Preliminary screening by AS and CC will determine the initial eligibility for the evaluation, using the information provided in the app summaries on the Apple App Store and Google Play Store. Apps that are deemed eligible will be purchased, and, if necessary, downloaded to an iOS device and Android device. Apps that, upon closer examination, do not meet the inclusion criteria will be excluded. Any disagreements between the reviewers will be discussed and, if necessary, settled by CC. All apps identified as being eligible for inclusion will be reviewed. An adapted PRISMA flow diagram will be used to record the details of the search, screening, and selection processes so that the evaluation can be reproduced.

#### App Evaluation

The quality of the apps will be evaluated using the MIND framework, which is based on the American Psychiatric Association’s app evaluation model. This framework includes 105 questions that assess various aspects of apps across 6 domains. The framework covers areas such as functionality, accessibility, inputs and outputs, privacy and security, data sharing, and evidence and clinical foundation. It also evaluates features and engagement styles. It helps assess app features, such as goal setting, psychoeducation, data tracking, cognitive behavioral therapy, and engagement elements, such as chat features and gamification [23,24]. MIND was chosen as an evaluation tool, as the platform offers richer data compared with other manual app rating frameworks. The literature highlights that digital navigators who use tools such as MIND, aid in the deployment of a more standardized approach to create lists of tested, approved, and authorized mood apps for clinicians to use and decrease barriers to app implementation in the clinic environment [25]. The list of data to be extracted to aid the quality evaluation is presented in Textbox 3.

**Full data charting list for app evaluation.**

**General**

- Name
- Year of launch
- Platform (iOS and Android)
- Cost
- Target population
- Number of downloads
- App store rating (1-5 stars)
- Clinical outcome (measurable change in health or quality of life)
- Evaluation measures (which evaluation measure is used)
- Published evaluation of app (yes or no)

**Features**

- Interface (use of audio, video, and images)
- Support (use of support buddy)
- Emergency (lifeline or SOS feature)
- Notifications (possibility to set different notifications on mobile device)
- Trends (possibility to display mood trends)
- Notes (possibility to take in-app notes)
- Reminders (possibility to set in-app reminders)
- Security (use of personal identification number protection to access the app)
- Information (possibility to access educational material)
- Research (option to use data for research purposes with permission)
- Data erasure (possibility of erasing full or partially selected data)
- Data export (possibility to export data including electronic health care records)

### Ethical Considerations

No ethical approval is required as exclusively secondary data will be used.

## Results

The literature search and screening began soon after the submission of this protocol in January 2024, and the review is expected to be completed by September 2024. As of September 2024, the app search and evaluation has begun and is expected to be completed and published by October 2024.

## Discussion

### Summary of Expected Findings

This review will provide an overview of the current landscape of mood apps targeted toward children, adolescents, and young people available in the market and the evidence on these mood apps. We will examine the apps’ features, usability, and scientific evidence base. The findings of this review will improve knowledge about not only the state of the literature on mood apps but also the quality of mood apps in active use and shed light on areas that require further research and improvement. The review will place a special emphasis on the end users of these mood apps, understanding the benefits of use for young people by examining the clinical base or publication of evidence of the apps. In addition, we will explore any interventions or strategies that have been used to enhance the quality and responsible development of mood apps.

### Implications and Comparison to Previous Work

By summarizing the existing features, strengths, and weaknesses of mood apps, as well as examining user experiences and satisfaction, we aim to contribute to the development of more effective and user-centered mood app frameworks. While previous research has analyzed the quality and literature on general apps for mental health, a similar synthesis about mood-monitoring apps is lacking [17,18,24]. We expect that the findings of this review will provide deeper insights into apps that specifically monitor mood and highlight evidence about digital self-monitoring of mood. The review will serve to inform the future development of mood-monitoring apps and, on a broader scale, enable a better understanding of the clinical use of regular mood monitoring for mental health management. In addition, we anticipate that this evaluation will play a crucial role in addressing ethical and usability concerns and promoting the responsible advancement of mood app technology.

### Limitations

The exclusion of literature published earlier than 3 years ago (ie, before 2021) is a potential limitation of phase 1 of this review. Although the time frame was chosen with the rapid evolution of technology in mind and enables a comprehensive analysis of the most recent evidence, we risk excluding relevant and good-quality apps evaluated earlier. Another limitation is that the exclusion of apps and studies not available in English may bias the findings of the review.

### Conclusions

The primary aims of this review are to provide an overview of the recent evidence on mood-monitoring apps and to assess the quality of the mood apps currently available in the marketplace and active use. Together, these analyses will help us understand the current landscape of mood apps targeted toward children, adolescents, and young people and potentially highlight the strengths and limitations of the research and the apps themselves. We expect that the outcomes of this review will inform the future development of mood apps for young people as well as scientific research into these apps. The results of the review will be disseminated through a peer-reviewed publication.

## Appendix

Multimedia Appendix 1 PRISMA (Preferred Reporting Items for Systematic Reviews and Meta-Analyses) checklist.

Multimedia Appendix 2 Search strings.

Multimedia Appendix 3 Clinical outcomes and measurement methods.

## Abbreviations

MARS: Mobile App Rating Scale
MIND: M-Health Index and Navigation Database
PICOS: Population, Intervention, Comparator, Outcome, Study
PRISMA: Preferred Reporting Items for Systematic Reviews and Meta-Analyses
PRISMA-ScR: Preferred Reporting Items for Systematic Reviews and Meta-Analyses extension for Scoping Reviews
