# Supplementary material for: Mental Health Monitoring for Young People Through Mood Apps: Protocol for a Scoping Review and Systematic Search in App Stores
Source: JMIR Res Protoc. 2024 Nov 19;13:e56400. doi: 10.2196/56400 (PMC11615542; doi:10.2196/56400)
Supplement: Multimedia Appendix 2 [file resprot_v13i1e56400_app2.docx]

**Table S1.**

| Database | Search String | Retrieved |
| --- | --- | --- |
| CINAHL | MH ( Mental Health OR Mental Disorders OR Psychological Well-Being OR Adolescent Health ) OR TI ( Mood OR depress* OR bipolar OR anxiety OR hypomania OR mania OR manic OR “mental health” OR anxiety OR anxious OR stress OR psychosis OR wellbeing OR well-being OR “adolescent health” OR “post-traumatic stress disorder*” OR PTSD ) OR AB ( Mood OR depress* OR bipolar OR anxiety OR hypomania OR mania OR manic OR “mental health” OR anxiety OR anxious OR stress OR psychosis OR wellbeing OR well-being OR “adolescent health” OR “post-traumatic stress disorder*” OR PTSD ) AND MH ( mobile applications OR Telemedicine ) OR TI ( app OR apps OR “mobile app*” OR “mhealth” OR telemedicine OR “mobile device*” OR smartphone OR “mobile phone” OR “mHealth” OR “mobile health” OR “health app” OR “digital app” OR telepsychology OR “digital intervention” OR “digital mental health intervention” OR “DMHI” ) OR AB ( app OR apps OR “mobile app*” OR “mhealth” OR telemedicine OR “mobile device*” OR smartphone OR “mobile phone” OR “mHealth” OR “mobile health” OR “health app” OR “digital app” OR telepsychology OR “digital intervention” OR “digital mental health intervention” OR “DMHI” ) AND MH ( Young Adult OR Paediatrics OR Adolescence OR Child ) OR TI ( “young adult” OR adolescen* OR teenage* OR students OR “young people” OR child* OR youth OR “young person" ) OR AB ( “young adult” OR adolescen* OR teenage* OR students OR “young people” OR child* OR youth OR “young person" ) | 2,036 |
| PubMed | ((((Mental Health OR Mental Disorders OR Psychological Well-Being OR Adolescent Health[MeSH Terms]) OR (mood[Title/Abstract] OR depress*[Title/Abstract] OR bipolar[Title/Abstract] OR anxiety[Title/Abstract] OR hypomania[Title/Abstract] OR mania[Title/Abstract] OR manic[Title/Abstract] OR "mental health"[Title/Abstract] OR anxious[Title/Abstract] OR stress[Title/Abstract] OR psychosis[Title/Abstract] OR wellbeing[Title/Abstract] OR well-being[Title/Abstract] OR "adolescent health"[Title/Abstract] OR "post-traumatic stress disorder*"[Title/Abstract] OR PTSD[Title/Abstract]))) AND (((Mobile Applications OR Telemedicine[MeSH Terms]) OR (app[Title/Abstract] OR apps[Title/Abstract] OR "mobile app*"[Title/Abstract] OR "mhealth"[Title/Abstract] OR telemedicine[Title/Abstract] OR "mobile device*"[Title/Abstract] OR smartphone[Title/Abstract] OR "mobile phone"[Title/Abstract] OR "mHealth"[Title/Abstract] OR "mobile health"[Title/Abstract] OR "health app"[Title/Abstract] OR "digital app"[Title/Abstract] OR telepsychology[Title/Abstract] OR "digital intervention"[Title/Abstract] OR "digital mental health intervention"[Title/Abstract] OR "DMHI"[Title/Abstract])))) AND (((Young Adult OR Pediatrics OR Adolescent OR Child[MeSH Terms]) OR ("young adult"[Title/Abstract] OR adolescen*[Title/Abstract] OR teenage*[Title/Abstract] OR students[Title/Abstract] OR "young people"[Title/Abstract] OR child*[Title/Abstract] OR youth[Title/Abstract] OR "young person"[Title/Abstract]))) | 7,203 |
| ACM digital library | [[Title: mood] OR [Title: depress*] OR [Title: bipolar] OR [Title: anxiety] OR [Title: hypomania] OR [Title: mania] OR [Title: manic] OR [Title: "mental health"] OR [Title: anxious] OR [Title: stress] OR [Title: psychosis] OR [Title: wellbeing] OR [Title: well-being] OR [Title: "adolescent health"] OR [Title: "post-traumatic stress disorder*"] OR [Title: ptsd] OR [Abstract: mood] OR [Abstract: depress*] OR [Abstract: bipolar] OR [Abstract: anxiety] OR [Abstract: hypomania] OR [Abstract: mania] OR [Abstract: manic] OR [Abstract: "mental health"] OR [Abstract: anxious] OR [Abstract: stress] OR [Abstract: psychosis] OR [Abstract: wellbeing] OR [Abstract: well-being] OR [Abstract: "adolescent health"] OR [Abstract: "post-traumatic stress disorder*"] OR [Abstract: ptsd]] AND [[Title: app] OR [Title: apps] OR [Title: "mobile app*"] OR [Title: "mhealth"] OR [Title: telemedicine] OR [Title: "mobile device*"] OR [Title: smartphone] OR [Title: "mobile phone"] OR [Title: "mhealth"] OR [Title: "mobile health"] OR [Title: "health app"] OR [Title: "digital app"] OR [Title: telepsychology] OR [Title: "digital intervention"] OR [Title: "digital mental health intervention"] OR [Title: "dmhi"] OR [Abstract: app] OR [Abstract: apps] OR [Abstract: "mobile app*"] OR [Abstract: "mhealth"] OR [Abstract: telemedicine] OR [Abstract: "mobile device*"] OR [Abstract: smartphone] OR [Abstract: "mobile phone"] OR [Abstract: "mhealth"] OR [Abstract: "mobile health"] OR [Abstract: "health app"] OR [Abstract: "digital app"] OR [Abstract: telepsychology] OR [Abstract: "digital intervention"] OR [Abstract: "digital mental health intervention"] OR [Abstract: "dmhi"]] AND [[Title: "young adult"] OR [Title: adolescen*] OR [Title: teenage*] OR [Title: students] OR [Title: "young people"] OR [Title: child*] OR [Title: youth] OR [Title: "young person"] OR [Abstract: "young adult"] OR [Abstract: adolescen*] OR [Abstract: teenage*] OR [Abstract: students] OR [Abstract: "young people"] OR [Abstract: child*] OR [Abstract: youth] OR [Abstract: "young person"]] | 1,772 |
| Springer* | (Mood OR depress* OR bipolar OR anxiety OR hypomania OR mania OR manic OR “mental health” OR anxious OR stress OR psychosis OR wellbeing OR well-being OR “adolescent health” OR“post-traumatic stress disorder*” OR PTSD) AND (app OR apps OR “mobile app*” OR “mhealth” OR telemedicine OR “mobile device*” OR smartphone OR “mobile phone” OR “mHealth” OR “mobile health” OR “health app” OR “digital app” OR telepsychology OR “digital intervention” OR “digital mental health intervention” OR “DMHI”) AND (“young adult” OR adolescen* OR teenage* OR students OR “young people” OR child* OR youth OR “young person”) | 844 |
| Scopus | ( TITLE-ABS-KEY ( mood OR depress* OR bipolar OR anxiety OR hypomania OR mania OR manic OR "mental health" OR anxious OR stress OR psychosis OR wellbeing OR well-being OR "adolescent health" OR "post-traumatic stress disorder*" OR ptsd ) AND TITLE-ABS-KEY ( app OR apps OR "mobile app*" OR "mhealth" OR telemedicine OR "mobile device*" OR smartphone OR "mobile phone" OR "mHealth" OR "mobile health" OR "health app" OR "digital app" OR telepsychology OR "digital intervention" OR "digital mental health intervention" OR "DMHI" ) AND TITLE-ABS-KEY ( "young adult" OR adolescen* OR teenage* OR students OR "young people" OR child* OR youth OR "young person" ) ) | 9,433 |
| EMBASE | ((exp mental health/ or exp mental disease/ or exp psychological well-being/ or exp adolescent health/) or (Mood or depress* or bipolar or anxiety or hypomania or mania or manic or "mental health" or anxiety or anxious or stress or psychosis or wellbeing or well-being or "adolescent health" or "post-traumatic stress disorder*" or PTSD).ab,kw,ti.) and ((exp mobile application/ or exp telemedicine/) or (app or apps or "mobile app*" or "mhealth" or telemedicine or "mobile device*" or smartphone or "mobile phone" or "mHealth" or "mobile health" or "health app" or "digital app" or telepsychology or "digital intervention" or "digital mental health intervention" or DMHI).ab,kw,ti.) and ((exp child/ or exp young adult/ or exp adolescence/ or exp pediatrics/) or ("young adult" or adolescen* or teenage* or students or "young people" or child* or youth or "young person").ab,kw,ti.) | 8795 |

*Only the keywords were searched, as the SpringerLink advanced search did not have an option to input MeSH terms; the results were filtered by date (2021-2024 inclusive) and reference type (articles) as the database caps the number of possible reference downloads to 1000 items maximum. These filters were applied to export the most relevant results as per our eligibility criteria.
