# Supplementary material for: Mental Health Monitoring for Young People Through Mood Apps: Protocol for a Scoping Review and Systematic Search in App Stores
Source: JMIR Res Protoc. 2024 Nov 19;13:e56400. doi: 10.2196/56400 (PMC11615542; doi:10.2196/56400)
Supplement: Multimedia Appendix 3 [file resprot_v13i1e56400_app3.docx]

**Table S1.**

| **Rating Scales** | |
| --- | --- |
| Bipolar Disorder | Duration and number of mood episodes |
|  | Mood Disorders Questionnaire |
| Mania/  Hypomania | Young Mania Rating Scales |
|  | Parent version of Young Mania Rating Scale (P-YMRS) |
|  | Manic State Rating Scale (MSRS) |
|  | Hypomania Checklist (HCL 32 and HCL 16) |
|  | Mania Diagnostic and Severity Scale (MADS) |
|  | Altman Self-Report Mania Rating Scale (ASRM) |
|  | Child Mania Rating Scale (CMRS) |
| Depression | Beck Depression Inventory (BDI) |
|  | Behavioural Activation for Depression Scale (BADS-SF) |
|  | Brief Psychiatric Rating Scale (BPRS) |
|  | Children's Depression Inventory (CDI) |
|  | Children's Depression Rating Scale (CDRS) |
|  | Clinically Useful Depression Outcome Scale (CUDOS) |
|  | Columbia Suicide Severity Rating Scale (C-SSRS) |
|  | Depression and Anxiety Stress Scales (DASS) |
|  | Depression Self-Rating Scale for Children |
|  | Edinburgh Postnatal Depression Scale |
|  | General Health Questionnaire |
|  | Geriatric Depression Scale (GDS) |
|  | Hamilton Rating Scale (HRSDD, HDRS, Ham-D) |
|  | HEADS-ED, used in hospital emergency departments |
|  | Hospital Anxiety and Depression Scale |
|  | Inventory of Depressive Symptomatology (IDS) |
|  | Kutcher Adolescent Depression Scale (KADS-11) |
|  | Major Depression Inventory (MDI) |
|  | Montgomery-Asberg Depression Scale (MADRS) |
|  | Mood and Feelings Questionnaire (MFQ) |
|  | Occupational Depression Inventory |
|  | Patient Health Questionnaire (PHQ-9) |
|  | Primary Care Evaluation of Mental Disorders (PRIME-MD) |
|  | Quick Inventory of Depressive Symptoms (QIDS) |
|  | Quick Inventory of Depressive Symptomatology Clinician (QIDS-C) |
|  | Quick Inventory of Depressive Symptomatology Self Report (QIDS-SR) |
| Measures of functioning and quality of life | |
|  | Clinical Global Impression-Bipolar (CGI-BP) |
|  | Clinical Global Impression- Improvement (CGI-I) |
|  | Clinical Global Impression- Severity (CGI-S) |
|  | Health of Nation Outcome Scales (HONOS) |
|  | Quality of Life Bipolar Disorder (QoL-BD) |
|  | EURO-QoL |
|  | EQ-5 DY |
|  | SF 60 |
|  | Mental health QoL |
|  | Global Assessment of Functioning |
|  | Social Adjustment Scale |
|  | WHODAS II |
|  | Life functioning questionnaire |
|  | WHOQOL BREF |
|  | Social Adaptation Self Evaluation Scale |
|  | Brief Quality of life in Bipolar Disorder Questionnaire |
|  | Work and Social Adjustment Scale |
|  | Functional Assessment Short Test |
|  | Social and Occupational Functioning Assessment Scale |
|  | Self-Reported Graphic Personal & Social Performance Scale (Bai Y.M. 2017) |
|  | Social and Occupational Functioning Assessment Scale (Olley 2005) |
|  | Bipolar Disorder Functioning Questionnaire (Ayedemir O. 2013) |
|  | Social Adjustment Scale - Modified version (Ball 2006) |
|  | Sheehan Disability Scale (Caroff S.M. 2018) |
|  | Functional Status Questionnaire (Medard E. 2010) |
|  | The KINDL Questionnaire (O'Donnell, Axelson et al. 2017) |
|  | The Longitudinal Interval Follow up Evaluation (Best 2017) |
|  | Social Functioning Scale (Saito S. 2016) |
|  | Heinrichs Quality of Life Scale (Ratheesh A. 2017) |
